# Metabolic Engineering of *Bacillus licheniformis* for Production of Acetoin

**DOI:** 10.3389/fbioe.2020.00125

**Published:** 2020-02-21

**Authors:** Chuanjuan Lü, Yongsheng Ge, Menghao Cao, Xiaoting Guo, Peihai Liu, Chao Gao, Ping Xu, Cuiqing Ma

**Affiliations:** ^1^State Key Laboratory of Microbial Technology, Shandong University, Jinan, China; ^2^State Key Laboratory of Microbial Metabolism, Joint International Research Laboratory of Metabolic and Developmental Sciences, School of Life Sciences and Biotechnology, Shanghai Jiao Tong University, Shanghai, China

**Keywords:** acetoin, *Bacillus licheniformis*, metabolic engineering, 2, 3-butanediol dehydrogenase, 2, 3-butanediol

## Abstract

Acetoin is a potential platform compound for a variety of chemicals. *Bacillus licheniformis* MW3, a thermophilic and generally regarded as safe (GRAS) microorganism, can produce 2,3-butanediol with a high concentration, yield, and productivity. In this study, *B. licheniformis* MW3 was metabolic engineered for acetoin production. After deleting two 2,3-butanediol dehydrogenases encoding genes *budC* and *gdh*, an engineered strain *B. licheniformis* MW3 (Δ*budC*Δ*gdh*) was constructed. Using fed-batch fermentation of *B. licheniformis* MW3 (Δ*budC*Δ*gdh*), 64.2 g/L acetoin was produced at a productivity of 2.378 g/[L h] and a yield of 0.412 g/g from 156 g/L glucose in 27 h. The fermentation process exhibited rather high productivity and yield of acetoin, indicating that *B. licheniformis* MW3 (Δ*budC*Δ*gdh*) might be a promising acetoin producer.

## Introduction

Acetoin (3-hydroxy-2-butanone) is widely used as a common additive in food industry and a building block for various chemicals (Liu et al., 2011; Xiao and Lu, 2014; Yang et al., 2017). It was listed as one of 30 platforms compound whose development and utilization was given priority by the US Department of Energy in 2004 (Xiao and Lu, 2014). Currently, commercially available acetoin is primarily produced from fossil feedstocks by chemical methods involving radical reactions and severe environmental pollution. With increasing concerns about production cost and environment protection, microbial fermentation production of acetoin has attracted extensive attention in recent years (Xiao and Lu, 2014; Yang et al., 2017).

Many native or recombinant microorganisms, including the genera *Serratia*, *Klebsiella*, *Saccharomyces*, *Gluconobacter*, and *Bacillus*, have abilities to produce acetoin (Sun et al., 2012; Wang et al., 2013, 2017; Bae et al., 2016; Jia et al., 2017). Among these species, *Bacillus* species which can produce various industrial products have become the research hotspot for acetoin production (Zhang Y. et al., 2013; Fan et al., 2018; Liu et al., 2019). Acetoin is an intermediate of 2,3-butanediol fermentation-pathway (Ji et al., 2011; Yang and Zhang, 2019). Except a few native acetoin producers, most of the *Bacillus* strains produce 2,3-butanediol but not acetoin as their major product (Fu et al., 2016; Dai et al., 2019). Thus, different metabolic engineering strategies were proposed to redistribute the carbon flux to acetoin in various *Bacillus* strains. For example, Zhang et al. successfully improved acetoin production by *Bacillus subtilis* through disruption of acetoin reductase (also called as 2,3-butanediol dehydrogenase) and expression of a water-forming NADH oxidase. Acetoin at a concentration of 56.7 g/L was produced with a yield of 0.378 g/g and a productivity of 0.639 g/[L h] (Zhang et al., 2014b).

*Bacillus licheniformis*, a generally regarded as safe (GRAS) strain with the characteristics of strong sugar consumption and fast growth rate, is a promising chassis strain for acetoin production. Natural *B. licheniformis* strains usually produce a mixture of (2*R*,3*R*)-2,3-butanediol and *meso*-2,3-butanediol isomers (Li L. et al., 2014; Qi et al., 2014; Qiu et al., 2016). In this study, two 2,3-butanediol dehydrogenases encoding genes in *B. licheniformis* MW3 were deleted and an engineered strain *B. licheniformis* MW3 (Δ*budC*Δ*gdh*) was constructed. The recombinant strain *B. licheniformis* MW3 (Δ*budC*Δ*gdh*) can produce 64.2 g/L acetoin within 27 h with a productivity of 2.378 g/[L h] and a yield of 0.412 g/g. Considering its desirable characteristics, *B. licheniformis* MW3 (Δ*budC*Δ*gdh*) may be a promising alternative for fermentative production of acetoin.

## Materials and Methods

### Enzymes and Chemicals

FastPfu DNA polymerase and T4 DNA ligase were purchased from TransGen Biotech (China) and Thermo Fisher (United States), respectively. Restriction enzymes were purchased from Thermo Fisher (United States). Polymerase chain reaction (PCR) primers were provided by Sangon (Shanghai, China). Racemic acetoin, diacetyl and 2,3-butanediol were purchased from Sigma. (2*R*,3*R*)-2,3-butanediol (98.0%), (2*S*,3*S*)-2,3-butanediol (99.0%), and *meso*-2,3-butanediol (98.0%) were purchased from ACROS (The Kingdom of Belgium). NADH was purchased from Roche (United States). Yeast extract (FM902) was a kind gift from Angel Yeast Co., Ltd. (Yichang, Hubei, China). All other chemicals were of analytical grade and commercially available.

### Bacterial Strains, Media, and Plasmids

All the strains and plasmids used in this study are listed in Supplementary Table S1. The plasmids pKVM1-Δ*budC* and pKVM1-Δ*gdh* based on the temperature sensitive plasmid pKVM1 were used for gene knockout in *B. licheniformis* MW3 (Waschkau et al., 2008; Rachinger et al., 2013; Ge et al., 2016). Lysogenic broth (LB) medium was used for cultivation of *Escherichia coli* and *B. licheniformi*s. NB medium (8 g/L Nutrient Broth, Difco) supplemented with 50 mM glucose was used for selection of *B. licheniformis* containing pKVM1. Ampicillin was used at a concentration of 100 μg/mL for the selection of *E. coli*. Erythromycin (5 μg/mL) and polymyxin B (40 μg/mL) were used for the selection of *B. licheniformis*. X-Gal was added at a concentration of 40 μg/mL for blue-white screening.

### Gene Knockout in *B. licheniformis* MW3

*E. coli* S17-1 λpir was used as donor strain to allow the conjugal transfer of plasmids pKVM1-Δ*budC* and pKVM1-Δ*gdh* into *B. licheniformis* MW3. After growth in liquid NB/glucose medium with erythromycin at 30°C, serial dilutions and spreading on NB/glucose plates with erythromycin and X-gal and incubation at 42°C, one of blue colonies of *B. licheniformis* MW3 with plasmid integration by homologous recombination was picked, inoculated in NB/glucose medium without antibiotic, and cultivated at 30°C for at least two passages. After dilutions and spreading on NB/glucose with X-gal, white colonies in which plasmids used to knockout were cured resulting from a second recombination event were picked. PCR was used to verify the disruption event of gene *budC* and *gdh* using primer pairs Δ*budC*-f/Δ*budC*-r and Δ*gdh*-f/Δ*gdh*-r (Supplementary Table S2), respectively.

### Enzyme Activity Assays

To measure 2,3-butanediol dehydrogenase activities in *B. licheniformis* MW3 and *B. licheniformis* MW3 (Δ*budC*Δ*gdh*), the cells of the strains were grown for 12 h, harvested, washed twice and resuspended in 67 mM phosphate buffer (pH 7.4), and disrupted by sonication with a Sonics sonicator (500 W; 20 kHz). Cell debris was removed through centrifugation (12,000 *g*, 15 min) and the resulting supernatants were used as the crude extracts for enzyme activity assays.

Activities of 2,3-butanediol dehydrogenase were spectrophotometrically assayed by measuring the change in absorbance at 340 nm corresponding to the oxidation of NADH or reduction of NAD^+^ at 30°C using a UV/visible spectrophotometer (Ultrospec 2100 pro, Amersham Biosciences, United States). For the reduction reaction, the reaction solution contains 0.2 mM of NADH and 5 mM of acetoin or diacetyl in 67 mM phosphate buffer (pH 7.4). For oxidation reactions, the reaction solution contains 10 mM (2*R*,3*R*)-2,3-butanediol, (2*S*,3*S*)-2,3-butanediol, and *meso*-2,3-butanediol and 1 mM NAD^+^ in 67 mM phosphate buffer (pH 7.4). One unit of activity was defined as the amount of enzyme that consumed or formed 1 μmol of NADH per min. The protein concentration in crude extract was measured by the Lowry method, with bovine serum albumin as the standard (Hartree, 1972).

### Batch and Fed-Batch Fermentations

*B. licheniformis* MW3 was maintained on LB agar slants at 4°C. A loop of cells from the fully grown slant was inoculated into 100 mL of LB in 500-mL Erlenmeyer flasks and incubated at 50°C on a rotary shaker at 180 r/min for 12 h to prepare the seed culture. Then, the seed culture was inoculated (5%, v/v) into the bioreactors for acetoin production.

Batch and fed-batch fermentations were conducted in a 1-L bioreactor (Infors AG, Bottmingen, Switzerland) with 0.8 L initial medium and a 5-L bioreactor (BIOSTAT B, B. Braun Biotech International GmbH, Germany) with 4 L initial medium, respectively. The seed culture was inoculated into the fermentation medium containing about 70 g/L glucose; 1 g/L triammonium citrate; 12 g/L yeast extract; 2 g/L K_2_HPO_4_⋅3H_2_O; 6.5 g/L sodium acetate; 0.25 g/L MgSO_4_⋅7H_2_O; 0.0225 g/L FeSO_4_⋅7H_2_O; 0.0075 g/L ZnSO_4_⋅7H_2_O; and 0.0038 g/L MnSO_4_⋅H_2_O. The cultivation was carried out at 50°C, stirring at 500 r/min, and airflow at 1.0 vvm (volume air per volume broth per minute). The pH was maintained at 7.0 by automatic addition of 6 M acetic acid and 6 M NaOH using a program-controlled peristaltic pump. Samples were collected periodically to determine the biomass, concentrations of glucose, 2,3-butanediol, and acetoin.

### Analytical Methods

Samples were centrifuged at 12,000 *g* for 10 min and the concentration of glucose was measured enzymatically using a bio-analyzer (SBA-40D, Shandong Academy of Sciences, China) after diluting to an appropriate concentration. The concentrations of 2,3-butanediol and acetoin were analyzed by gas chromatography (GC; GC2014c, Shimadzu) with capillary GC columns (AT. SE-54, inside diameter, 0.32 mm; length, 30 m; Chromatographic Technology Center, Lanzhou Institute of Chemical Physics, China) as described previously (Zhang L. et al., 2016).

## Results and Discussion

### Construction of the Strain *B. licheniformis* MW3 (Δ*budC*Δ*gdh*)

**FIGURE 1:**
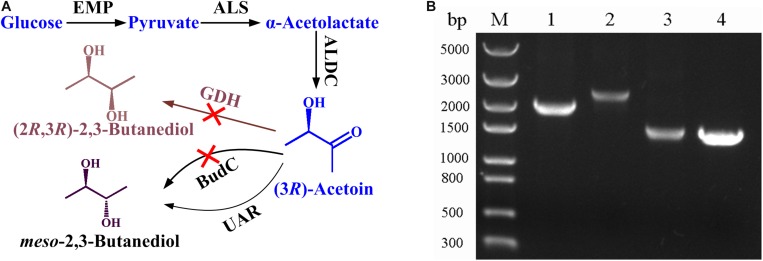
Construction of *B. licheniformis* MW3 (Δ*budC*Δ*gdh*) for acetoin production. **(A)** Technology roadmap for acetoin production from glucose by *B. licheniformis* MW3 (Δ*budC*Δ*gdh*). ALS, α-acetolactate synthase; ALDC, α-acetolactate decarboxylase; BudC, *meso*-2,3-butanediol dehydrogenase; GDH, (2*R*,3*R*)-2,3-butanediol dehydrogenase; UAR, unidentified acetoin reductase (based on the results of gas chromatograph in Supplementary Figure S1, this enzyme produces *meso*-2,3-butanediol as the reduction product of acetoin). **(B)** Analysis of PCR fragments to confirm *budC* and *gdh* disruption. Lane M: molecular mass standard (DNA/*Hin*dIII); Lane 1: PCR product of *budC* amplified with *B. licheniformis* MW3 genomic DNA as the template; Lane 2: PCR product of *gdh* amplified with *B. licheniformis* MW3 genomic DNA as the template; Lane 3: PCR product of *budC* amplified with *B. licheniformis* MW3 (Δ*budC*Δ*gdh*) genomic DNA as the template; Lane 4: PCR product of *gdh* amplified with *B. licheniformis* MW3 (Δ*budC*Δ*gdh*) genomic DNA as the template. The PCRs were performed with primer pairs Δ*budC*-f/Δ*budC*-r and Δ*gdh*-f/Δ*gdh*-r, respectively.

*B. licheniformis* MW3 is a GRAS and thermophilic strain which can efficiently produce *meso*-2,3-butanediol and (2*R*,3*R*)-2,3-butanediol with a simple stereoisomer formation mechanism (Ge et al., 2016). As shown in Figure 1A, glucose is converted to pyruvate by glycolytic pathway, then α-acetolactate synthase (ALS) condenses two molecules of pyruvate to generate one molecules of α-acetolactate, and then α-acetolactate is transformed into one molecules of acetoin by α-acetolactate decarboxylase (ALDC). Two stereospecific 2,3-butanediol dehydrogenases, (2*R*,3*R*)-2,3-butanediol dehydrogenase (encoded by *gdh*) and *meso*-2,3-butanediol dehydrogenase (encoded by *budC*), are responsible for the production of (2*R*,3*R*)-2,3-butanediol and *meso*-2,3-butanediol, respectively (Ge et al., 2016). To construct an acetoin producing strain based on *B. licheniformis* MW3, both (2*R*,3*R*)-2,3-butanediol dehydrogenase and *meso*-2,3-butanediol dehydrogenase encoding genes should be deleted. The temperature sensitive plasmid pKVM1 was utilized as described previously. The mutant strain *B. licheniformis* MW3 (Δ*budC*Δ*gdh*) was selected and the result in Figure 1B shows that the PCR using the primer pairs generated products of the expected sizes.

### Activities of 2,3-Butanediol Dehydrogenases in *B. licheniformis* MW3 (Δ*budC*Δ*gdh*)

The activities of (2*R*,3*R*)-2,3-butanediol dehydrogenase and *meso*-2,3-butanediol dehydrogenase in *B. licheniformis* MW3 (Δ*budC*Δ*gdh*) were assayed. As shown in Supplementary Table S3, (2*S*,3*S*)-2,3-butanediol oxidation activity was rather low in both *B. licheniformis* MW3 and *B. licheniformis* MW3 (Δ*budC*Δ*gdh*). Both (2*R*,3*R*)-2,3-butanediol and *meso*-2,3-butanediol oxidation activities decreased in *B. licheniformis* MW3 (Δ*budC*Δ*gdh*). Especially, decrease of acetoin reduction activity occurred after deletion of (2*R*,3*R*)-2,3-butanediol dehydrogenase and *meso*-2,3-butanediol dehydrogenase encoding genes. Taking advantage of its low acetoin reduction activity, acetoin produced by ALDC might not be reduced to 2,3-butanediol and the strain *B. licheniformis* MW3 (Δ*budC*Δ*gdh*) was used for acetoin production in the subsequent experiments.

### Batch Fermentation of Acetoin by *B. licheniformis* MW3 (Δ*budC*Δ*gdh*)

**FIGURE 2:**
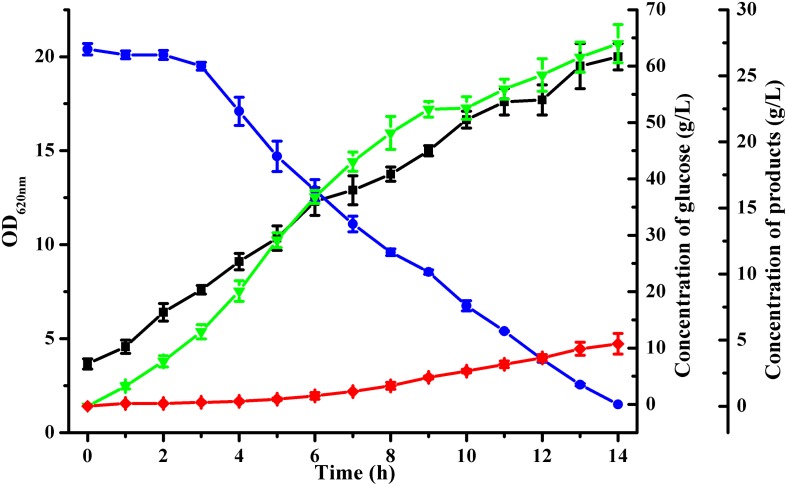
Time-course of batch fermentation by *B. licheniformis* MW3 (Δ*budC*Δ*gdh*). 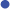, Glucose; 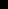, OD_620nm_; 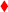, 2,3-butanediol; 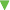, acetoin. The experiment was conducted in a 1-L bioreactor (Infors AG, Bottmingen, Switzerland) with 0.8 L initial medium with an initial glucose concentration of 70 g/L approximately. Cultivation was carried out at 50°C and an initial pH of 7.0. The pH was maintained at 7.0 by automatic addition of 6 M acetic acid and 6 M NaOH. The agitation speed was 500 r/min and the aeration rate was 1 vvm. Data were the means ± SDs from three parallel experiments.

Batch fermentation was conducted in a 5-L bioreactor with 4 L initial medium to analyze acetoin production by *B. licheniformis* MW3 (Δ*budC*Δ*gdh*). As shown in Figure 2, inactivation of both *budC* and *gdh* led to a high acetoin accumulation in *B. licheniformis* MW3 (Δ*budC*Δ*gdh*). This strain consumed 63 ± 1 g/L glucose in 14 h and produced 27.4 ± 1.45 g/L acetoin, with a yield of 0.435 ± 0.023 g/g (Figure 2). Although the growth of *B. licheniformis* MW3 (Δ*budC*Δ*gdh*) was slightly lower than that of *B. licheniformis* MW3, this strain can produce acetoin instead of 2,3-butanediol as its major product (Supplementary Table S4).

### Fed-Batch Fermentation of Acetoin by *B. licheniformis* MW3 (Δ*budC*Δ*gdh*)

**FIGURE 3:**
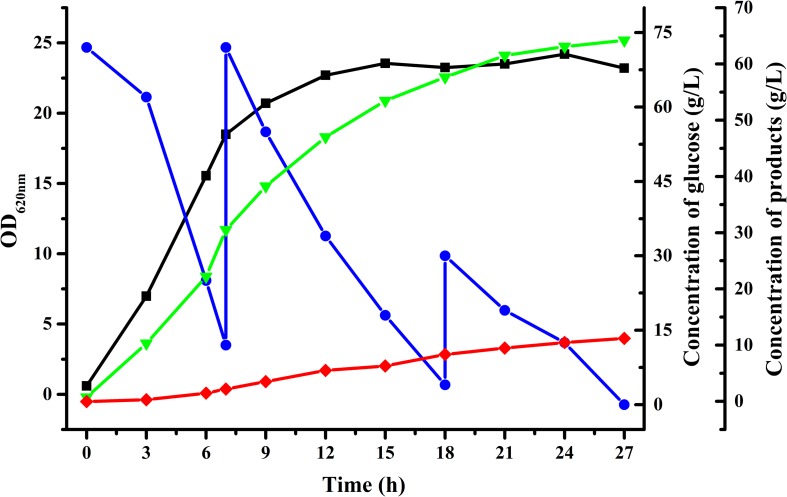
Time-course of fed-batch fermentation by *B. licheniformis* MW3 (Δ*budC*Δ*gdh*). 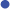, Glucose; 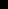, OD_620nm_; 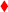, 2,3-butanediol; 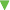, acetoin. The experiment was conducted in a 5-L bioreactor (BIOSTAT B, B. Braun Biotech International GmbH, Germany) with 4 L initial medium with an initial glucose concentration of 70 g/L approximately. Cultivation was carried out at 50°C and an initial pH of 7.0. The pH was maintained at 7.0 by automatic addition of 6 M acetic acid and 6 M NaOH. The agitation speed was 500 r/min and the aeration rate was 1 vvm. The fed-batch fermentation was conducted by feeding glucose solution when the residual glucose in the fermentation was lower than 10 g/L. Data were from a representative fed-batch fermentation repeated for three times.

To achieve higher acetoin concentrations, fed-batch fermentation was carried out with an initial glucose concentration of about 70 g/L using *B. licheniformis* MW3 (Δ*budC*Δ*gdh*). As shown in Figure 3, 64.2 g/L acetoin from 156 g/L glucose was obtained in 27 h by *B. licheniformis* MW3 (Δ*budC*Δ*gdh*). The yield of acetoin was 84.1% of the theoretical value and the average productivity was 2.378 g/[L h]. The carbon flux channeled into the acetoin biosynthetic might be further enhanced since there were still 2,3-butanediol accumulated during the fermentation (Figure 3 and Supplementary Figure S1). Toxicity of acetoin can hinder its higher production by various microorganisms. It was reported that an approximate 30% cell growth of *B. licheniformis* will be inhibited with 40 g/L exogenous acetoin (Yuan et al., 2019). Thus, production of 2,3-butanediol may be due to induction of an unidentified acetoin reductase or *meso*-2,3-butanediol dehydrogenase to resistant the toxicity of acetoin at high concentrations. Higher production of acetoin by *B. licheniformis* may be accomplished by searching the undiscovered acetoin reductase or overexpressing NADH oxidase, which could lead to prevention of NADH-dependent reduction of acetoin.

Several recombinant *Bacillus* strains have been used to produce acetoin (Table 1). Based on a two-stage pH control strategy, the group of Li obtained a high acetoin concentration of 73.6 g/L with a productivity of 0.77 g/[L h] using metabolically engineered *B. subtilis* overexpressing 2,3-butanediol dehydrogenase (Zhang et al., 2014a). Fan et al. successfully improved the coproduction of uridine and acetoin by modification of acetoin metabolism in engineered *B. subtilis*. The recombinant strain can produce 60.48 g/L acetoin and 40.62 g/L uridine after 48 h of fed-batch fermentation (Fan et al., 2018). The group of Chen isolated a *B. licheniformis* strain WX-02 which can produce γ-poly-glutamic acid accompanied with 2,3-butanediol (Li X. et al., 2014). *B. licheniformis* WX-02Δ*budC*Δ*acoR* was constructed through deleting *budC* and acetoin degradation transcriptional activator encoding gene *acoR*. Fed-batch fermentation through a three-stage agitation strategy during which 2,3-butanediol was first accumulated and then converted to acetoin using *B. licheniformis* WX-02Δ*budC*Δ*acoR* can produce 78.79 g/L acetoin with a yield of 0.31 g/g and a productivity of 0.58 g/[L h] at 37°C (Li et al., 2017). *B. licheniformis* MW3 is a thermophilic strain which can produce 2,3-butanediol as its major fermentation product with higher yield and productivity than that of *B. licheniformis* WX-02 at 50°C, implying a latent capacity of strain MW3 for efficient acetoin production (Ge et al., 2016; Qiu et al., 2016). Thus, *B. licheniformis* MW3 (Δ*budC*Δ*gdh*) was constructed and the recombinant strain was able to produce 64.2 g/L acetoin with a relatively high productivity (2.378 g/[L h]) through a simple fermentation process. The values of productivity and yield of acetoin produced using *B. licheniformis* MW3 (Δ*budC*Δ*gdh*) were the highest ever obtained in acetoin production by recombinant *Bacillus* species. *B. licheniformis* MW3 (Δ*budC*Δ*gdh*) might be useful for the production of acetoin on a commercial scale.

**TABLE 1:** Fermentation production of acetoin by recombinant *Bacillus* species.

**Strain**	**Engineering strategy**	**Concentration (g/L)**	**Yield (g/g)**	**Productivity (g/[L h])**	**References**
*B. subtilis* F126-2	Disrupting 2,3-butanediol dehydrogenase gene *bdhA* and overexpression of *alsSD* operon based on a uridine producing strain	60.48	/	1.26	Fan et al., 2018
*B. subtilis* BSA	Over-expression of 2,3-butanediol dehydrogenase in *B. subtilis* JNA 3-10, applying a two-stage pH control strategy	73.6	0.408	0.77	Zhang et al., 2014a
*B. subtilis* BMN	Moderate-expression of the water-forming NADH oxidase C in *bdhA* disrupted *B. subtilis* JNA 3-10	56.7	0.675	0.639	Zhang et al., 2014b
*B. subtilis* 168/pMA5-zwf	Over-expression of glucose-6-phosphate dehydrogenase G6PDH in *B. subtilis* 168	43.3	0.33	0.36	Bao et al., 2015
*B. subtilis* BS168D	Blocking of 2,3-butanediol dehydrogenase gene *bdhA* in *B. subtilis* 168	24.6	0.246	0.342	Zhang et al., 2017
*B. subtilis* ZB02	Over-expression of xylose transport protein AraE, xylose isomerase XylA, and xylulokinase XylB in *B. subtilis* 168ARSRCPΔ*acoA*Δ*bdhA*, using glucose, xylose, and arabinose as substrates	62.2	NR^a^	0.864	Zhang B. et al., 2016
*B. subtilis* BSK814A4	Deleting *araR*, *bdhA*, *acoA* and inserting a native *xyl* operon into genome-reduced *B. subtilis* strain BSK814	23.3	0.46	0.194	Yan et al., 2018
*B. subtilis* BSUW06	Overexpressing *alsSD* operon and deleting *bdhA*, *acoA*, and *pta* in *B. subtilis* 168	19.8	NR	0.566	Wang et al., 2012
*B. subtilis* PAR	Moderate enhancement of ALsR expression using promoter P_*bdhA*_ in *B. subtilis* 168	41.5	0.35	0.43	Zhang X. et al., 2013
*B. licheniformis* WX-02Δ*budC*Δ*acoR*	Deleting *budC* and acetoin degradation transcriptional activator encoding gene *acoR* in *B. licheniformis* WX-02	78.79	0.31	0.58	Li et al., 2017
*B. licheniformis* MW3 (Δ*budC*Δ*gdh*)	Deleting *budC* and *gdh* in *B. licheniformis* MW3	64.4	0.412	2.378	This work

*^*a*^NR, not reported.*

## Conclusion

We constructed a recombinant strain *B. licheniformis* MW3 (Δ*budC*Δ*gdh*) for acetoin production. Acetoin at a high concentration (64.2 g/L) was produced with a productivity of 2.378 g/[L h] and a yield of 0.412 g/g through fed-batch fermentation by *B. licheniformis* MW3 (Δ*budC*Δ*gdh*). Because of its GRAS characteristic and ability to produce acetoin with high concentration, productivity, and yield, this strain should be a promising alternative for the practical production of acetoin.

## Data Availability Statement

The raw data supporting the conclusions of this article will be made available by the authors, without undue reservation, to any qualified researcher.

## Author Contributions

CL, YG, and MC performed experiments. CL, CG, and CM wrote the manuscript and conceived the study. CL, YG, MC, XG, and PL were involved in analysis and interpretation of experimental data. CM and PX coordinated the project.

## Conflict of Interest

The authors declare that the research was conducted in the absence of any commercial or financial relationships that could be construed as a potential conflict of interest.
