# Supplementary material for: Metabolic Engineering of Bacillus licheniformis for Production of Acetoin
Source: Front Bioeng Biotechnol. 2020 Feb 21;8:125. doi: 10.3389/fbioe.2020.00125 (PMC7047894; doi:10.3389/fbioe.2020.00125)
Supplement: Supplementary file 1 [file Data_Sheet_1.docx]

**Table S1** Bacterial strains and plasmids used in this work.

| Name | Characteristic ^a^ | Reference or source |
| --- | --- | --- |
| Strain |  |  |
| *E. coli* DH5α | F^-^, φ80d lacZΔM15, Δ(lacZYA-argF)U169, recA1, endA1, hsdR17(rk^-^, mk^+^), phoA, supE44λ^-^, thi^-1^, gyrA96, relA1 | Novagen |
| *E. coli* S17-1 λpir | *TpR SmR recA, thi, pro, hsdR-M+RP4: 2-Tc:Mu: Km Tn7 λpir.* | Wang et al., 2014 |
| *B. licheniformis* MW3 | *B. licheniformis* DSM13 (Δ*hsdR1*Δ*hsdR2*) | Waschkau et al., 2008 |
| *B. licheniformis* MW3 (Δ*budC*Δ*gdh*) | *B. licheniformis* MW3 mutant obtained by deletion of both *budC* and *gdh* | This work |
| Plasmid |  |  |
| pKVM1 | Suicide plasmid for gene knockout, oriT, traJ, Amp^r^, Em^r^ | Rachinger et al., 2013 |
| pKVM1-Δ*gdh* | Δ*gdh* was cloned into pKVM1 | Ge et al., 2016 |
| pKVM1-Δ*budC* | Δ*budC* was cloned into pKVM1 | Ge et al., 2016 |

^a^ Kan^r^, kanamycin resistant; Amp^r^, ampicillin resistant; Em^r^, erythromycin resistant.

**Table S2** Primers used in this work.

| **Primer ^a^** | **Sequence (5’–3’)** | **Use** |
| --- | --- | --- |
| Δ*budC*-f | AAACCATGGAATAAACGAGTTGACGGAAA | Amplification of a 1920 bp fragment containing *budC* with its upstream and downstream sequences  Amplification of a 1920 bp fragment containing *budC* with its upstream and downstream sequences |
| Δ*budC*-r | TTTGGATCCTATGCTCGCGGTGTTCTAT |  |
| Δ*gdh*-f | ATTTAGATCTAACAAGCCGCGTCATTCAAG | Amplification of a 2241 bp fragment containing *gdh* with its upstream and downstream sequences |
| Δ*gdh*-r | TACCGTGGATCCGCTTTAAG | Amplification of a 2241 bp fragment containing *gdh* with its upstream and downstream sequences |

^a^ “f” of primer name means the sense primer; “r” of primer name means antisense primers. These primers are outside of the 5’ and 3’ flanking regions of the genes on the plasmids and only used to verify the disruption event of gene *budC* and *gdh* in genome of white colonies resulting from a double crossover event with cured knockout plasmids.

**Table S3** Specific activity of the 2,3-butanediol dehydrogenase in *B. licheniformis* MW3 and *B. licheniformis* MW3 (Δ*budC*Δ*gdh*)

| Substrate | Specific activity (U/mg) | |
| --- | --- | --- |
|  | MW3^c^ | MW3 (Δ*budC*Δ*gdh*)^d^ |
| Oxidation^a^ |  |  |
| (2*R*,3*R*)-2,3-butanediol | 0.058 ± 0.019 | 0.004 ± 0.001 |
| *meso*-2,3-butanediol | 0.463 ± 0.058 | 0.003 ± 0.000 |
| (2*S*,3*S*)-2,3-butanediol | 0.004 ± 0.001 | 0.001 ± 0.001 |
| Reduction^b^ |  |  |
| Racemic acetoin | 0.628 ± 0.003 | 0.002 ± 0.001 |
| Diacetyl | 0.135 ± 0.010 | 0.003 ± 0.001 |

^a^ Assay conditions: the reaction systems contain 67 mM phosphate buffer (pH 7.4), 1 mM NAD^+^ and 10 mM substrates.

^b^ Assay conditions: the reaction systems contain 67 mM phosphate buffer (pH 7.4), 0.2 mM NADH and 5 mM substrate.

^c^ Data from Ge et al., 2016.

^d^ Data were the means ± SDs from three parallel experiments.

**Table S4** Batch fermentation performance of *B. licheniformis* MW3 and *B. licheniformis* MW3 (△*budC*Δ*gdh*)^a^

|  | MW3^c^ | MW3 (Δ*budC*Δ*gdh*)^d^ |
| --- | --- | --- |
| Glucose (g/L) | 70 ± 2 | 63 ± 1 |
| OD_620nm_ | 21.9 ± 0.5 | 20 ± 0.7 |
| Acetoin (g/L) | 2.30 ± 0.22 | 27.43 ± 1.45 |
| 2,3-Butanediol (g/L) | 33.34 ± 2.15 | 4.72 ± 0.78 |
| Yield (g/g)^b^ | 0.033 ± 0.003 | 0.435 ± 0.023 |

^a^ The seed cultures of the different strains were inoculated into 1-L bioreactor containing 0.8 L initial medium and incubated at 50°C with stirring at 500 rpm, and airflow at 1.0 vvm. Then, biomass, glucose consumption, 2,3-butanediol and acetoin production by different strains were assayed.

^b^ Yield was calculated based on the acetoin production.

^c^ Data from Ge et al., 2016.

^d^ Data were the means ± SDs from three parallel experiments.


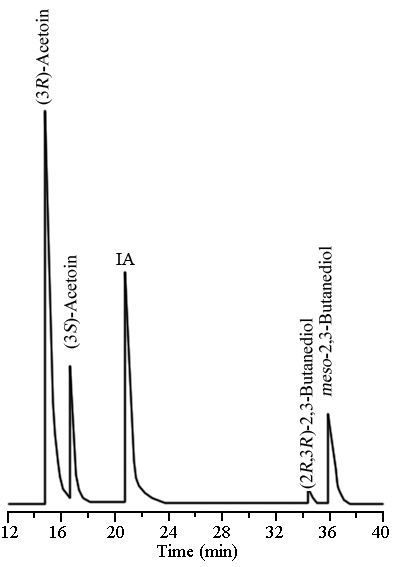


**FIGURE S1 |** Gas chromatography analysis of the acetoin and 2,3-butanediol produced by strain *B. licheniformis* MW3 (△*budC*Δ*gdh*). IA, isoamyl alcohol was used as the internal standard.
